# Prevalence and Diversity of Haemotropic *Mycoplasma* Species in Cats and Their Ectoparasites (Fleas and Ticks)

**DOI:** 10.3390/vetsci11020081

**Published:** 2024-02-09

**Authors:** Miglė Razgūnaitė, Indrė Lipatova, Algimantas Paulauskas, Justina Snegiriovaitė, Birutė Karvelienė, Gintaras Zamokas, Monika Laukutė, Jana Radzijevskaja

**Affiliations:** 1 Department of Biology, Vytautas Magnus University, K. Donelaičio Str. 58, LT-44248 Kaunas, Lithuania; migle.razgunaite@vdu.lt (M.R.); indre.lipatova@vdu.lt (I.L.); algimantas.paulauskas@vdu.lt (A.P.); justina.snegiriovaite@vdu.lt (J.S.); monika.laukute@vdu.lt (M.L.); 2 Faculty of Veterinary Medicine, Lithuanian University of Health Sciences, Tilžės Str. 18, LT-47181 Kaunas, Lithuania; birute.karveliene@lsmu.lt (B.K.); gintaras.zamokas@lsmu.lt (G.Z.)

**Keywords:** *Mycoplasma haemofelis*, ‘*Candidatus* Mycoplasma haematominutum’, domestic cats, *Ctenocephalides felis*, *16S rRNA* gene

## Abstract

**Simple Summary:**

*Mycoplasma* spp. are bacteria that can cause various infections in different animal species. In cats, bacteria have been described as haemotropic and non-haemotropic. The haemotropic *Mycoplasma* spp. parasitizes red blood cells and can induce haemolytic anemia. In Lithuania, the diagnosis of *Mycoplasma* infection in cats in veterinarian clinics is based on the microscopic analysis of blood smears revealing pathogens on the surface of the erythrocytes. However, in cases of low bacteremia and due to the morphological similarity between species, the microscopic analysis of haemoplasma is complicated and could lead to incorrect diagnosis. This shows the necessity to use molecular analysis for haemoplasma diagnosis.

**Abstract:**

*Mycoplasma* spp. pathogens frequently cause chronic and acute diseases in cats. The aim of the present study was to investigate the presence and genetic diversity of *Mycoplasma* spp. in cats and their ectoparasites using PCR and sequence analysis of the *16S rRNA* gene. Blood samples were collected from 541 domestic and stray cats in Lithuania. Ectoparasites (153 fleas and 321 ticks) were collected from owned domestic cats that live both outdoors and indoors. *Mycoplasma* spp. were detected in 7.2% of cat blood samples and 4.4% of *Ctenocephalides felis* fleas. The sequence analysis revealed the presence of *Mycoplasma haemofelis* in 1.1% of cats and ‘*Candidatus* Mycoplasma haematominutum’ in 4.8% of cats. *Ct. felis* fleas harboured *M. haemofelis*. To the best of the authors’ knowledge, this is the first report on the prevalence and molecular characterisation of *Mycoplasma* bacteria in cats in Lithuania and cat fleas in the Baltic States.

## 1. Introduction

*Mycoplasma* spp. bacteria can cause various infections in animal species [1]. In cats, haemotropic and non-haemotropic types of mycoplasmas have been described. The haemotropic *Mycoplasma* spp. parasitize red blood cells and can induce haemolytic anaemia [2]. The three worldwide distributed haemoplasma species known to infect cats are *Mycoplasma haemofelis*, ‘*Candidatus* Mycoplasma haematominutum’ and ‘*Candidatus* Mycoplasma turicensis’ [3]. In addition, another species ‘*Candidatus* Mycoplasma haematoparvum’-like, was detected in cats in the USA, Chile, and Portugal [4,5,6].

Feline haemoplasma infections are usually subclinical, but after a longer period of time, due to weakened immunity, a wide range of clinical signs may appear, including anaemia, pyrexia, lethargy, and splenomegaly [2,6,7]. *Mycoplasma haemofelis* is more pathogenic than ‘*Ca*. M. haematominutum’ and ‘*Ca*. M. turicensis’ [2]. Although ‘*Ca*. M. haematominutum’ and ‘*Ca*. M. turicensis’ are less pathogenic, they can result in disease in immunocompromised cats. The untreated disease may result in death. *Mycoplasma* between cats can be transmitted via infected blood transfusion or during aggressive interaction between cats [6,8]. Ectoparasites, such as fleas and ticks, still very commonly infest cats and, therefore, can also play an important role in the transmission route [6,7,9].

Feline haemoplasma infections are usually subclinical, but after a longer period of time, due to weakened immunity, a wide range of clinical signs may appear, including anaemia, pyrexia, lethargy, and splenomegaly [3,7,8]. *M. haemofelis* is more pathogenic than ‘*Ca*. M. haematominutum’ and ‘*Ca*. M. turicensis’ [3]. Although ‘*Ca*. M. haematominutum’ and ‘*Ca*. M. turicensis’ are less pathogenic, they can result in disease in immunocompromised cats. The untreated disease may result in death [9,10]. *Mycoplasma* between cats can be transmitted via infected blood transfusion or during aggressive interaction between cats [7,11]. Ectoparasites, such as fleas and ticks, still very commonly infest cats and, therefore, can also play an important role in the transmission route [7,8,12].

In Lithuania, the diagnosis of *Mycoplasma* infection in cats in veterinarian clinics is based on the microscopic analysis of blood smears revealing pathogens on the surface of the erythrocytes. However, in cases of low bacteremia and due to the morphological similarity between species, the microscopic analysis of haemoplasma is complicated and could lead to incorrect diagnosis [3]. Also, the untrained eye may fail to distinguish strain precipitate and other blood artifacts from haemoplasma. Blood culture has long been a classic method for detecting pathogenic microorganisms in the bloodstream, serving as the gold standard for isolating such microorganisms in bloodstream infections. However, haemoplasmas, being fastidious bacteria, are challenging to grow on cell-free media. Due to this inability to cultivate haemoplasmas in vitro, molecular techniques have become the gold standard for diagnosing infections, investigating their prevalence, and describing new species [13,14]. Polymerase chain reaction (PCR) assays have emerged as the preferred diagnostic method for detecting haemoplasma infections, offering superior sensitivity and specificity compared to traditional cytological methods [15]. However, it is important to note that false-negative results may occur, mainly in the presence of low bacteremia in blood [2,16]. Despite an intense immune response and antibiotic treatment, cats often remain asymptomatic pathogen carriers [3]. The use of molecular techniques is necessary to detect and identify *Mycoplasma* species effectively. The aim of the present study was to investigate the presence and diversity of *Mycoplasma* spp. in cats using PCR amplification and sequence analysis based on the *16S rRNA* gene. A further objective of this work was to assess the prevalence of *Mycoplasma* spp. in fleas and ticks collected from owned domestic cats. To date, no data on the prevalence and diversity of *Mycoplasma* species in cats and their ectoparasites have been documented in Lithuania.

## 2. Materials and Methods

### 2.1. Domestic Cats Blood Collection

A total of 541 blood samples from owned cats (n = 523) and shelter cats (n = 18) were collected in two veterinary clinics and one shelter located in Kaunas (central Lithuania) and one veterinary clinic in Klaipėda (western Lithuania) during 2016–2021. Cats were presented to veterinary clinics for prophylactic examination or due to various illnesses. Blood samples were taken in the clinics for diagnostic purposes; no additional blood was drawn for the haemoplasma analysis. The age, gender, and clinical symptoms of the cats were recorded. Cats were divided into two age groups: young cats < 1 year old (n = 42) and adults > 1 year old (n = 499). Based on their health status during the clinic visit, which was determined through physical and morphological examinations, cats were divided into apparently healthy (n = 249) and unhealthy (n = 292) (Table 1). Information about outdoor access or the flea infestation status of cats was not available. Feline blood samples were taken from the cephalic vein into EDTA-containing tubes and kept at −20 °C until DNA extraction.

### 2.2. Ectoparasite Sample Collection

Cats whose blood was collected in veterinary clinics did not have ectoparasites during the clinic visit. All shelter animals received treatment for internal and external parasites and were not infested at the time of sample collection. Due to this, additional ectoparasite sample collection was carried out by asking owners to collect ectoparasites from their pets and provide them for research.

Ectoparasites (fleas and ticks) were collected from owned domestic cats (that live outdoors and indoors) in 2015–2021. Blood specimens were not available from these cats. Ectoparasites collected from each cat were placed in separate 1.5 mL tubes with 70% ethanol and kept at +4 °C until investigation. Flea and tick species were identified based on morphological criteria [17,18]. 

### 2.3. DNA Extraction and PCR Amplification

Following the manufacturer’s instructions, DNA was extracted from EDTA blood using a GeneJet Whole Blood Genomic DNA Purification Kit (Thermo Fisher Scientific, Vilnius, Lithuania). DNA from engorged ticks was extracted from each specimen individually using a Genomic DNA Purification Kit (Thermo Fisher Scientific, Vilnius, Lithuania) according to the manufacturer’s instructions. DNA from non-engorged ticks and fleas was extracted individually from each specimen using 2.5% ammonium hydroxide [19].

Conventional PCR targeting a 600 bp region of the *16S rRNA* gene of *Mycoplasma* spp. was performed using the primers 322s (5′-GCCCATATTCCTACGGGAAGCAGCAGT-3′) and 938as (5′-CTCCACCACTTGTTCAGGTCCCCGTC-3′) created by Varanat et al. [20]. The conventional PCR amplifications were carried out in a 25 µL final volume consisting of 2 µL of extracted DNA, 5 µL 5× MyTaq reaction buffer (Bioline Reagents Ltd., London, UK), 1 µL M of each primer, and 0.2 µL of MyTaq DNA Polymerase (1U) (Bioline Reagents Ltd., London, UK). Amplification reaction was performed as follows: initial denaturation at 95 °C for 2 min, 35 cycles of denaturation at 94 °C for 20 s, annealing at 68 °C for 25 s, and extension at 72 °C for 30 s and a final extension step at 72 °C for 3 min. In each PCR run, a negative control (consisting of sterile, double-distilled water added to the PCR mix rather than DNA) and positive control (DNA of *Mycoplasma* positive cat, infection confirmed by blood smear and real-time PCR) were used.

PCR products were visualized on a 1.5% agarose gel (Thermo Fisher Scientific, Vilnius, Lithuania). *Mycoplasma*-positive samples selected for DNA sequencing were purified using the GeneJET™ Gel Extraction Kit (Thermo Fisher Scientific, Vilnius, Lithuania) and sequenced (Macrogen Europe company, Amsterdam, The Netherlands). The acquired sequences were examined using the Mega11 (Molecular Evolutionary Genetics Analysis Version 11) software program [21] and aligned using the ClustalW and BLAST computer algorithms with each other and the previously released sequences in GenBank. The the Maximum-likelihood (ML), Tamura 3-parameter model and 1000 bootstrap repeats were used to create a phylogenetic tree.

The partial *16S rRNA* gene sequences for representative samples obtained in this study were submitted to the GenBank database under the accession numbers: OQ355649 and from OQ361729 to OQ361734 for *M. haemofelis* and from OQ361735 to OQ361760 for ‘*Ca*. M. haematominutum’.

### 2.4. Statistical Analysis

Descriptive statistical analysis was performed using Statistica for Windows (version 7.0, StatSoft, Tulsa, OK, USA) with 95% confidence intervals (CI 95%) to compare the prevalence of *Mycoplasma* in cats of different ages, sex, health status, lifestyle, and cat ectoparasites. The observed differences were considered to be significant when *p* < 0.05. 

### 2.5. Clinical Infection Cases

A total of 689 cats were referred for consultation and diagnostics at a Small Animal Teaching Hospital in Kaunas from 2019 to 2022. Among them, 547 cats underwent morphological and biochemical blood tests. Blood samples were taken for diagnostic purposes, all cats showed clinical signs of various diseases. All blood samples had been anticoagulated with ethylenediamine tetra-acetic acid (EDTA). All morphological blood tests were done with an “IDEXX LaserCyte” haematology blood analyser.

A total of 167 cats had cytological tests on blood smears. Blood smears were selected to be performed for cats with outdoor access, which had previously had ectoparasites, were lethargic, had lost weight, had symptoms of depression, fever, or jaundice or had been diagnosed with other feline infectious diseases, and for cats with anaemia and lymphocytosis on morphological blood tests. Blood smear cytology was chosen as an additional diagnostic method.

**Figure 1:**
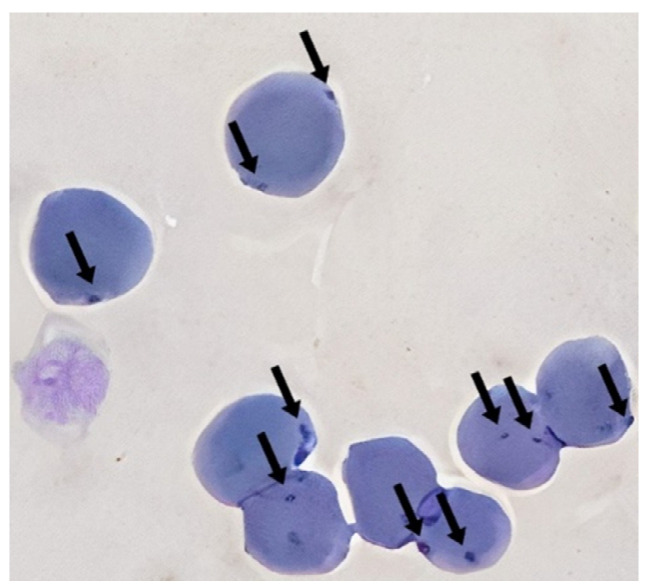
Modified Wright–Giemsa staining of feline blood smear showing small coccoid dot shaped (Arrow) on RBCs surface, which later was confirmed as *Mycoplasma* sp. by RT-PCR.

The blood smear was subjected to Wright–Giemsa staining and cytologically assessed for the presence of haemoplasma organisms (Figure 1). The stained samples were stored at room temperature. Cytological evaluation of blood smears was performed using an “Olympus BX43” microscope.

## 3. Results

*Mycoplasma* DNA was detected in 7.2% (39/541) of cat blood samples (Table 1). All Mycoplasma-positive cats were adults. The prevalence of *Mycoplasma* spp. was significantly higher in shelter cats (22.2%; 4/18) compared to owned cats (6.7%; 35/523) (χ^2^ = 6.274, *p* < 0.05). It is important to acknowledge that there is a substantial difference in the sample sizes between clinics and shelters, which could potentially impact the statistical significance. A chi-square test of independence showed that there was no significant association between gender (χ^2^ = 0.191, *p* > 0.05) or health status (χ^2^ = 0.1, *p* > 0.05) and infection with *Mycoplasma* spp.

A total of 153 fleas representing three species (137 *Ctenocephalides felis*, 15 *Ctenocephalides canis*, and one *Nosopsyllus fasciatus*) were collected from 28 owned domestic cats in four Lithuanian districts (Alytus, Kaunas, Klaipėda, and Panevėžys). The intensity of flea infestation ranged from 1 to 34 fleas per cat. 

A total of 321 ticks representing two species, Dermacentor reticulatus (n = 55) and Ixodes ricinus (n = 266), were collected from 59 cats in seven Lithuanian districts (Alytus, Kaunas, Klaipėda, Marijampolė, Tauragė, Utena, Vilnius) (Table 2). The intensity of tick infestation ranged from one to 16 ticks per cat. 

Six flea samples collected from two cats and one *I. ricinus* male tick sample were positive (3.9% (6/153) and 0.3% (1/321), respectively) after PCR amplification of the *16S rRNA* gene. *Mycoplasma* DNA was detected only in fleas belonging to the *Ct. felis* species (4.4%; 6/137). Four specimens were females (4.1%; 4/98) and two males (5.1%; 2/39). Infection rate of *Mycoplasma* in fleas per cat varied from 11.8% to 16.7% (Table 3). There was no statistical significance in the prevalence of infection between the sexes of fleas (χ^2^ = 0.073, *p* > 0.05) (Table 4). 

**Figure 2:**
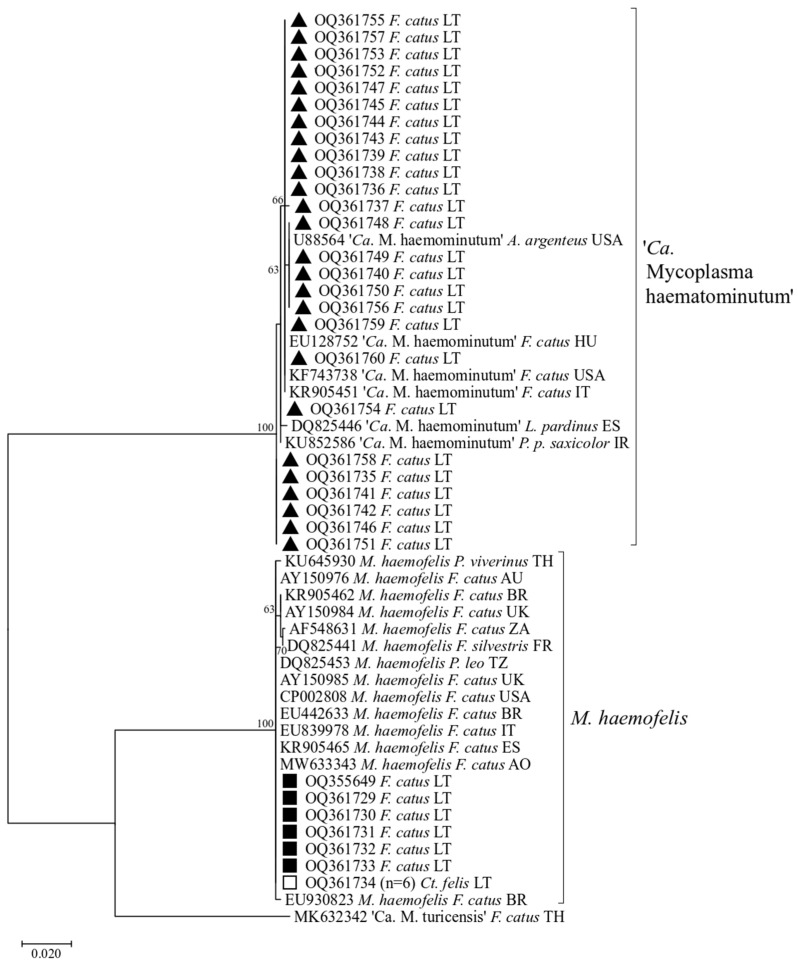
Phylogenetic tree of the *16S rRNA* gene of *Mycoplasma* spp. inferred using the maximum-likelihood method and Tamura 3-parameter model with 1000 bootstrapping replications. Samples sequenced in the present study are marked with ■ (*M. haemofelis* from *F. catus*), ▲ (‘*Ca.* M. haematominutum’ from *F. catus*) and □ (*M. haemofelis* from *Ct. felis*). In parentheses (n x) is the number of samples that the sequence represents.

A total of 39 good-quality PCR products derived from cats (n = 32) and cat ectoparasites (six from fleas and one from a tick) were sequenced and analysed. Sequence analysis demonstrated that six cats and six *Ct. felis* fleas were infected with *M. haemofelis* and 26 cats with ‘*Ca.* M. haematominutum’ (Figure 2, Table 5). The *16S rRNA* gene fragment obtained from *I. ricinus* tick showed a high similarity (99.03%) to the obligate intracellular bacterium Rickettsiella (GenBank accession no. KT697666).

A total of 54 *Mycoplasma* spp. *16S rRNA* gene sequences (33 from this study and 21 sequences derived from the GenBank database) were included in phylogenetic analysis. The ‘*Ca.* M. turicensis’ (GenBank accession no. MK632342) was chosen as the outgroup. The phylogenetic tree showed two well-supported clusters (Figure 2). One cluster contained M. haemofelis, and another consisted of ‘Ca. M. haematominutum’ sequences (Table 5). 

Sequences of *M. haemofelis* obtained from both cats and fleas in the current investigation demonstrated a clear 100% match in their *16S rRNA* gene sequences. These sequences also exhibited complete conformity with counterpart sequences from diverse geographical origins such as Angola (GenBank accession no. MW633343), Australia (GenBank accession no. AY150976), Brazil (GenBank accession no. EU442633), Italy (GenBank accession no. EU839978), Spain (GenBank accession no. KR905465), United Kingdom (GenBank accession no. AY150985), USA (GenBank accession no. CP002808), and Tanzania (GenBank accession no. DQ825453) (Figure 2; Table 6). However, upon scrutiny of other sequences sourced from GenBank, subtle variations were observed at four specific nucleotide positions (GenBank accession no. KR905462, AY150984, DQ825441, KU645930, EU930823, AF548631).

Within the Lithuanian isolates of ‘*Ca.* M. haematominutum’, five sequence variants characterized by variable nucleotides were identified (Table 7). Thirteen sequences (sequence variant I) derived from Lithuanian specimens were found to exhibit 100% *16S rRNA* gene sequence identity with corresponding ‘*Ca.* M. haematominutum’ sequences available in the GenBank database, previously identified in domestic cats originating from Hungary (GenBank accession no. EU128752), the USA (GenBank accession no. KF743738), and Italy (GenBank accession no. KR905451). Five sequences (sequence variant II) displayed complete identity (100%) with ‘*Ca.* M. haematominutum’ isolate from Apodemus argenteus rodent from the USA (GenBank accession no. U88564), differing from preceding sequences by a single nucleotide substitution (A→G) at position 337 within the analysed sequences. Six sequences (sequence variant III) were 100% identical to the *16S rRNA* gene sequences were mutually identical but differed from sequences available in Genbank by two nucleotide substitutions (T→G) at position 78 and (C→T) at position 456). One ‘*Ca.* M. haematominutum’ *16S rRNA* sequence (sequence variant IV) acquired in this study exhibited uniqueness by diverging from others by a single nucleotide substitution (C→T) at position 333. One sequence (sequence variant V) was 100% identical to the *16S rRNA* gene sequence obtained in Panthera pardus saxicolor from Iran (GenBank accession no. KU852586), contrasting with prior sequences by one nucleotide substitution (C→T) at position 456 within the analysed sequences.

### Clinical Infection Cases

In Lithuania, there has been a notable increase in diagnosed cases of feline haemotrophic mycoplasmosis over the last five years, prompting veterinary attention. A total of 54 (32%; 54/167) confirmed cases of *Mycoplasma* infection in cats detected during 2019–2022 in Kaunas were analysed in the Small Animal Teaching Hospital. The diagnosis was based on the examination of blood smears stained with the Wright–Giemsa stain. 

Eleven samples underwent subsequent confirmation through real-time PCR testing. Eight of these samples were selected due to abnormal blood smear results, while the remaining three were from cases showing relapse. Additional validation was conducted through real-time PCR targeting the *16S rRNA* gene. The samples were sent to LABOKLIN GMBH & CO.KG in Germany. It is important to note that this additional testing was carried out only for pets whose owners consented to the extra analysis.

Haemoplasmas were detected at the commercial laboratory, and we were not able to perform phylogenetic analysis of the sequences at the time of the clinical diseases. Out of the 54 cases, 74% (40/54) of the infected cats exhibited haemolytic anaemia, 83% (45/54) displayed lethargy, 89% (48/54) experienced weight loss, 13% (7/54) showed signs of jaundice, and 91% (49/54) had elevated body temperature and 7% (4/54) of the cats did not manifest any clinical symptoms. After the definitive diagnosis of haemoplasma infection, cats were treated with oral doxycycline (10 mg/kg/day PO q24h for 21 days) as this is known as the primary choice for the treatment of haemoplasma infections [4,22,23]. Supplementary immunosuppression treatment with prednisolone (1 mg/kg PO q24h) was applied to 31% (17/54) of cats, and 35% of cats (19/54) received haemotransfusion due to extremely advanced autoimmune anaemia. Notably, three cats that had tested positive for *Mycoplasma* spp. by PCR relapsed into infectious haemolytic anaemia around eleven days after completing the doxycycline treatment course. The presence of haemoplasma was once again detected in the blood smears of the relapsed cats. This observation aligns with earlier research findings, which demonstrated that despite robust immune responses and antibiotic therapy, cats frequently persist as asymptomatic carriers of this pathogen [2,3].

## 4. Discussion

To the best of the authors’ knowledge, this is the first report on the prevalence and genetic diversity of *Mycoplasma* bacteria in cats and cat fleas in Lithuania. The present study showed the usefulness of molecular methods not only for the identification of *Mycoplasma* species and genotypes but also in cases when symptoms were weak or non-specific and could not provide sufficient diagnostic information to a veterinarian. 

The amplification of feline haemoplasma DNA from the cat blood samples confirms the presence of two feline haemoplasma species—*M. haemofelis* and ‘*Ca*. M. haematominutum’. Overall, the prevalence of feline haemoplasma infections found in this study (7.2%) was considerably similar to that obtained in other studies (7.8% in Spain; 9.4% in Germany; 8.9% in Brazil) [7,24,25]. The most prevalent haemoplasma species in the Lithuanian cat population was ‘*Ca*. M. haematominutum’ (4.8%), whereas *M. haemofelis* was less common (1.1%). Other studies have also shown a higher prevalence of ‘*Ca*. M. haematominutum’ compared with *M. haemofelis* [7,25,26,27,28]. The least prevalent among the three feline haemoplasmas, ‘*Ca*. M. turicensis’, was not detected in the present study. Previous studies have also reported a low prevalence of this haemoplasma species (0–2%) [7,26,27,28,29].

Our study showed high prevalence of haemoplasma infections not only in unhealthy (with various clinical symptoms) cats but also in apparently healthy cats (7.5% and 6.8%, respectively). *Mycoplasma* bacteria are opportunistic pathogens, and, although the prevalence of these pathogens is high in feline populations, infection with them rarely causes clinical symptoms [3,30]. Commonly observed symptoms attributed to *Mycoplasma* infection in cats include lethargy, anorexia, depression, dehydration, and altered general blood test parameters [22]. Our findings showed that 17 out of 39 infected cats did not have any clinical symptoms (Table 1). The observed clinical symptoms in other cats were not specific and presented by lethargy, diarrhoea, visual impairment, and changes in general blood test parameters, which are characteristic of a wide range of diseases [31,32]. In the current study, three out of six cases of *M. haemofelis* infection exhibited clinical symptoms, all of which were consistent with previously described symptoms in *Mycoplasma*-positive cats [2,3,6]. All *M. haemofelis* sequences obtained in this study were identical to each other, suggesting no differences between the genetics of the pathogen and its clinical manifestation in cats. Additionally, in this study, the only cat with a confirmed case of anaemia was infected with ‘*Ca*. M. haematominutum’ rather than *M. haemofelis*, which is typically more associated with anaemia cases. However, cases of anaemia have been reported in which only ‘*Ca*. M. haematominutum’ infection was diagnosed, just like in this study [33]. 

As of now, complete genomes of seven isolates of *M. haemofelis* have been documented, encompassing strains from Japan (GenBank accession no. AP022325), Brazil (GenBank accession no. CP114890, CP114889, CP115656, CP114888), and Canada (GenBank accession no. CP103988, CP103993, CP110269), including multiple assemblies from different isolates. Additionally, there is a complete genome of ‘*Ca*. M. haematominutum’ from the United Kingdom (GenBank accession no. HE613254). Earlier studies have reported varying prevalence rates of haemoplasmas in domestic cats across different regions, such as Thailand (22.9%) [34], Brazil (36.4%) [10] and Latvia (17.2%) [28]. A recent phylogenetic analysis conducted by Berzina et al. (2021) based on *16S rRNA* gene fragment sequences from the GenBank indicated the widespread occurrence of *M. haemofelis* in pet cats and wild animals globally, with analysed sequences belonging to 10 different sequence variants based on the variation of nucleotides in 11 positions [28]. Similarly, ‘*Ca*. M. haematominutum’ *16S rRNA* gene sequences exhibited eight variants, with sequences originating from various locations worldwide [28]. Interestingly, sequence variants for both species did not exhibit specific geographic tendencies. 

In our study, the *16S rRNA* gene analyses showed that III sequence variant (Table 7) grouped six ‘*Ca*. M. haematominutum’-positive cases from which only one had clinical symptoms (GenBank accession no. OQ361751). This observation highlights the complexity of the relationship between genetic variants of ‘*Ca*. M. haematominutum’ and the manifestation of clinical symptoms suggest that factors beyond the *16S rRNA* genetic identity may contribute to the variability in clinical outcomes. Furthermore, phylogenetic analysis revealed a possibility of ‘*Ca*. M. haematominutum’ inter-animal transmission in positive shelter cats, as two of four positive cats had the same genetic variant II (GenBank accession no. OQ361748, OQ361750).

The precise mode of transmission of *Mycoplasma* pathogens among cats remains unresolved. However, the potential involvement of an arthropod vector gains support from the identification of feline haemoplasma DNA in fleas and ticks obtained from cats and/or their surroundings [35,36]. In the present study, 4.4% of *Ct. felis* fleas collected from two owned domestic cats were positive for *M. haemofelis*. *Mycoplasma* DNA in cat fleas has also been previously reported in various countries [12,35,37,38,39,40,41,42,43]. A previous study [44] conducted in Malaysia showed that most of the *Mycoplasma*-infected cats were heavily infested with ectoparasites—81.7% were infected with fleas, 56.7% with lice and 30% with mites. These findings suggest the possible transmission route of feline mycoplasma pathogens. It has long been suspected that transmission of these organisms has been via the bite of infected fleas [3,43]. Woods et al. [39] experimentally demonstrated that fleas can be a possible vector for *M. haemofelis*. In contrast, other experimental studies reported that *M. haemofelis* could not be transmitted through *Ct. felis* flea bites [40,41]. *Mycoplasma* DNA was not detected in cat fleas in a study performed by Azrizal-Wahid et al. (2021) in Malaysia [42]. Despite this, cat fleas are still considered to play a role in *Mycoplasma* transmission [3,44].

In the present study, none of the examined *I. ricinus* and *D. reticulatus* ticks were found positive for *Mycoplasma* spp. Similarly, to our results, other studies have also shown that ticks collected from cats were not infected with *Mycoplasma* spp. [35,45,46]. However, the fact that *Mycoplasma* DNA has not been detected in ticks collected from cats does not prove the incompetence of this vector to transmit this pathogen. In other studies, *Mycoplasma* DNA has been detected by real-time PCR in 0.6% *I. ricinus* and *Ixodes trianguliceps* ticks collected from cats in the UK [36] and in 1.2% *Ixodes* sp. and *Rhipicephalus sanguineus* ticks from cats in Switzerland [32]. It could be suggested that infections of Ixodidae ticks with these bacteria are rare, or that the amount of the pathogens is extremely low and difficult to detect using conventional PCR. The sample size may also influence the results. Our study demonstrated that other pathogenic bacteria can be detected using the *16S rRNA* gene for the amplification of *Mycoplasma* DNA. The primer used in this study [20] allowed us to identify the DNA of bacteria closely related to an obligate intracellular *Rickettsiella* bacterium in one specimen of *I. ricinus*. This bacterial endosymbiont is widespread in arthropods, including Ixodidae ticks. It has been established that there are at least four species: *Rickettsiella popilliae*, *R. grylli*, *R. chironomi*, and *R. stethorae* [47,48]. Some are pathogenic to arthropods, and some are transovarially transmitted endosymbionts [47,49]. 

### Clinical Infection Cases

The clinical infection cases underscore the diverse and often significant clinical manifestations observed in cats diagnosed with haemoplasma infection, including haemolytic anaemia, lethargy, jaundice, pyrexia, and more. The study sheds light on treatment challenges, emphasizing the necessity for supplementary interventions in some instances and noting the occurrence of relapses even after completing the standard treatment course. Clinical infection cases also highlighted the need for PCR when the microscopic analysis of haemoplasma is complicated, particularly in cases of low bacteremia where accurate diagnosis may be challenging and could lead to incorrect diagnosis [3], demonstrating superior sensitivity and specificity compared to traditional cytological methods [15]. These findings significantly contribute to understanding the clinical management and outcomes associated with feline haemoplasma infections.

## 5. Conclusions

To the best of the authors’ knowledge, this is the first report on the prevalence and molecular characterisation of *Mycoplasma* bacteria in cats and cat fleas in Lithuania. Results highlight the need for PCR use when the microscopic analysis of haemoplasma is complicated. Highlighting the necessity of molecular analysis to accurately diagnose haemotropic mycoplasmosis in cats and provide knowledge for a better understanding of the epidemiology of haemoplasmosis, which will help to ensure effective therapy and develop a disease control program in Lithuania.

## Figures and Tables

**Table 1:** Characteristics of the tested feline samples: lifestyle, sex, age, and health status of all cats testing positive or negative for haemoplasma.

Characteristics of Tested Cats	N	*Mycoplasma*-Positive Cats	Odds Ratio	95% CI	*p*
shelter cats	18	4	3.98	1.25–12.74	*p* = 0.01
owned cats	523	35	1		
male	287	22	1.16	0.6–2.23	*p* = 0.66 *
female	254	17	1		
young kittens < 1 year old	42	0	-	-	-
adults > 1 year old	499	39	-		
healthy	249	17	0.9	0.47–1.73	*p* = 0.75 *
unhealthy	292	22	1		

Abbreviations: N, number of tested cats; CI, confidence interval; *, statistically not significant.

**Table 2:** Detection of *Mycoplasma* in ticks infesting cats.

Ticks	Sex	N
*I. ricinus*	♂	24
	♀	242
*D. reticulatus*	♂	21
	♀	34
Total		321

Abbreviations: ♂, male; ♀, female; N, number of tested samples.

**Table 3:** Number of mycoplasma-positive flea infestations in cats.

Cats	No of *Mycoplasma* Positive/No of Tested Fleas (%)
1	4/34 (11.8%)
2	2/12 (16.7%)

**Table 4:** Detection of *Mycoplasma* in fleas infesting ticks.

Fleas	Sex	N	*Mycoplasma*-Positive Fleas	Odds Ratio	95% CI	χ^2^	*p*	MIR (%)
*Ct. felis*	♂	27	2	2.12 1	0.37–12.23	0.736	*p* = 0.39 *	4.379
	♀	110	4					
*Ct. canis*	♂	4	0	-	-	-	-	-
	♀	11	0					
*N. fasciatus*	♂	0	0	-	-	-	-	-
	♀	1	0					
Total		153	6					

Abbreviations: ♂, male; ♀, female; N, number of tested samples; CI, confidence interval; χ^2^, Chi-square value; *, statistically not significant; MIR, minimum infection rate.

**Table 5:** *Mycoplasma* species detected in cats and cat ectoparasites.

	*Mycoplasma* spp.	*M. haemofelis*	‘*Ca.* M. haematominutum’
shelter cats	4	0	4
owned cats	35	6	22
Total in cats	39	6	26
*Ct. felis* fleas	6	6	0

**Table 6:** Nucleotide variations in the *16S rRNA* gene sequences between Lithuanian and foreign strains of *M. haemofelis*. Sequences identified in this study are in bold.

GenBank Accession Numbers	Nucleotide Positions				Geographic Origin
	40	114	206	543	
*Felis catus* AY150976, AY150985, EU839978, CP002808, EU442633, KR905465, MW633343; *Panthera leo* DQ825453; *Felis catus* **OQ355649**, **OQ361729**, **OQ361730**, **OQ361731**, **OQ361732**, **OQ361733**; *Ctenocephalides felis* **OQ361734** (n = 6)	T	A	T	C	Australia, United Kingdom, Italy, United States of America, Brazil, Spain, Angola, Tanzania, Lithuania
*Felis catus* KR905462, AY150984; *Felis silvestris* DQ825441	.	.	.	T	Brazil, United Kingdom, France
*Prionailurus viverinus* KU645930	C	.	.	.	Thailand
*Felis catus* EU930823	.	.	C	.	Brazil
*Felis catus* AF548631	.	C	.	T	South Africa

**Table 7:** Nucleotide variations in the *16S rRNA* gene sequences between Lithuanian and foreign strains of ‘*Ca.* M. haematominutum’. Sequences detected in this study are bolded.

GenBank Accession Numbers	Sequence Variants	Nucleotide Positions					Geographic Origin
		78	222	333	337	456	
*Felis catus* EU128752, KF743738, KR905451, **OQ361736**, **OQ361738**, **OQ361739**, **OQ361743**, **OQ361744**, **OQ361745**, **OQ361747**, **OQ361752**, **OQ361753**, **OQ361755**, **OQ361757**, **OQ361759**, **OQ361760**	I	T	T	C	A	C	Hungary, United States of America, Italy, Lithuania
*Apodemus argenteus* U88564; *Felis catus* **OQ361740**, **OQ361748**, **OQ361749**, **OQ361750**, **OQ361756**	II	.	.	.	G	.	United States of America, Lithuania
*Felis catus* **OQ361735**, **OQ361741**, **OQ361742**, **OQ361746**, **OQ361751**, **OQ361758**	III	G	.	.	.	T	Lithuania
*Felis catus* **OQ361737**	IV	.	.	T	.	.	Lithuania
*Panthera pardus saxicolor* KU852586; *Felis catus* **OQ361754**	V	.	.	.	.	T	Iran, Lithuania

## Data Availability

Data is contained within the article.
